# Successful Treatment of *Candida auris* Ventriculitis With Intravenous Liposomal Amphotericin B and Oral Flucytosine: A Case Report

**DOI:** 10.1093/ofid/ofae743

**Published:** 2024-12-20

**Authors:** Nayoung Kang, Victor Yu-Ching Hsu, Charles Christopher Bailey

**Affiliations:** Pharmacy Department, Providence St Joseph Hospital, Orange, California, USA; Medicine Department, Providence St Joseph Hospital, Orange, California, USA; Medicine Department, Providence St Joseph Hospital, Orange, California, USA

**Keywords:** antifungal therapy, *Candida auris*, flucytosine, liposomal amphotericin B, ventriculitis

## Abstract

*Candida auris* is a rapidly emerging fungal pathogen associated with high resistance rates, particularly in healthcare settings. It most commonly affects patients with severe underlying medical conditions and requiring complex medical care. Patients with invasive medical devices tend to be at increased risk for getting *C auris* and developing infection. This article presents a case of *C auris* ventriculitis successfully treated with intravenous liposomal amphotericin B and oral flucytosine. A 41-year-old man with multiple comorbidities, including recent placement of a ventriculoperitoneal shunt, presented with suspected sepsis. *Candida auris* was isolated from cerebrospinal fluid cultures. Antifungal therapy along with removal of the shunt led to resolution of infection without complications. This case highlights the challenges posed by *C auris* infections and underscores the importance of appropriate treatment strategies.

## Teaser

This case report contributes to the understanding of effective treatment strategies for *Candida auris* ventriculitis, highlighting the role of liposomal amphotericin B and flucytosine in achieving favorable outcomes without resorting to intraventricular administration of antifungal agents.

*Candida auris* is a fungal pathogen associated with nosocomial infections, particularly in patients with complex medical conditions and invasive devices. Its frequent multidrug resistance presents challenges in treatment, with echinocandins often recommended as first-line therapy for most infections [1, 2]. However, central nervous system (CNS) infections, such as ventriculitis, pose additional challenges due to variable and often poor penetration of available antifungal agents. This report describes a case of *C auris* ventriculitis successfully managed with liposomal amphotericin B and flucytosine.

## CASE REPORT

A 41-year-old Asian man with a recent history of right basal ganglia hemorrhage and ventriculoperitoneal shunt placement was brought to the emergency department from the skilled nursing facility (SNF) to which he had been discharged a day earlier, with tachycardia, fever, and suspected sepsis. He exhibited residual left hemiparesis but remained alert and responsive to commands. A computed tomography (CT) scan of the head without contrast demonstrated slightly increased size of the ventricles since his scan a week prior. A CT scan of the abdomen and pelvis also demonstrated new or worsening subcapsular fluid collection in the left hepatic lobe. Neurosurgery was consulted for further input regarding these findings.

*Candida auris* polymerase chain reaction (PCR) screening was performed on a nares/axillae swab per our routine practice for patients admitted from an SNF or long-term acute care facility (LTACF) and was positive consistent with *C auris* colonization. Cerebrospinal fluid (CSF) cultures from an aspiration of the shunt reservoir and a lumbar puncture came back positive with yeast; based on this information, empiric coverage that would include *C auris* was initiated. An empiric antifungal regimen of liposomal amphotericin B (5 mg/kg intravenously every 24 hours) and flucytosine (25 mg/kg/dose per oral 4 times daily) was started.

On hospital day 3, the ventriculoperitoneal shunt was removed, an external ventricular drain was placed, and a subgaleal fluid collection was aspirated. Cultures of the shunt and subgaleal fluid obtained during these procedures yielded growth of *C auris*. Microscan sensitivities from the initial cultures were available at this time (Table 1). According to Centers for Disease Control and Prevention (CDC)–defined breakpoints (Table 2), based on those established for closely related *Candida* species and on expert opinion, our *C auris* isolates were susceptible to echinocandins and amphotericin but resistant to fluconazole. Therefore, therapy with liposomal amphotericin B and flucytosine, which had been begun empirically, was continued.

**Table 1.** Patient's Cerebrospinal Fluid Culture Susceptibility

Antibiotic	MIC, g/mL
Anidulafungin	0.12
Micafungin	0.06
Caspofungin acetate	0.12
Flucytosine	≤0.06
Voriconazole	0.5
Itraconazole	0.12
Fluconazole	256
Amphotericin B	1
Posaconazole	0.03

Abbreviation: MIC, minimum inhibitory concentration.

**Table 2.** Centers for Disease Control and Prevention *Candida auris* Breakpoints

Antibiotic	Tentative MIC Breakpoints, g/mL
Fluconazole	>32
Voriconazole and other second-generation triazoles	Consider using fluconazole susceptibility as a surrogate. However, isolates that are resistant to fluconazole may respond to other triazoles occasionally.
Amphotericin B	≥2
Anidulafungin	≥4

Abbreviation: MIC, minimum inhibitory concentration.

Repeat blood and CSF cultures collected 4 days after the removal of the ventriculoperitoneal shunt were negative for fungal growth, as were 8 more samples obtained over the subsequent 2 months. The extraventricular drain was removed on hospital day 11. A new ventriculoperitoneal shunt was placed 2 weeks later with ongoing negative CSF cultures and a decline in pleocytosis from 38 to 1 cells/μL.

Liposomal amphotericin B and flucytosine were continued until the intraoperative CSF smear and culture were confirmed negative (Table 3), completing a total of 4 weeks of antifungal therapy. While on liposomal amphotericin B, the patient received pre- and posthydration infusions with 500 mL sodium chloride 0.9% with dextrose flushes in between, which might have helped with preventing an acute kidney injury. When the patient was discharged back to an SNF, he was alert and following commands and no longer had leukocytosis, serum creatinine was at baseline, and infectious markers were stable.

**Table 3.** Cerebrospinal Fluid Culture Reports

Day Since Admission	Culture Type	Results
Day 1	CSF, lumbar puncture	*Candida auris*
Day 3 (day 2 of antifungal treatment)	Shunt	*C auris*
	Brain, lateral ventricle, right	*C auris*
	CSF, subgaleal	*C auris*
Day 4	Shunt	Negative
Day 7	Shunt	Negative
Day 11	Shunt	Negative
Day 14	Shunt	Negative

Abbreviation: CSF, cerebrospinal fluid.

## DISCUSSION

*Candida* CNS infection can present as a manifestation of disseminated candidiasis, in the presence of ventricular drainage devices, or as isolated chronic meningitis [3, 4]. In this case, the infection was associated with a ventriculoperitoneal shunt. According to the Infectious Diseases Society of America (IDSA) practice guidelines for ventriculitis, fever without an identifiable source of infection may suggest a CSF shunt infection, as observed in our patient [5]. Furthermore, the guidelines emphasize that CSF cultures are critical for diagnosing healthcare-associated ventriculitis and recommend culturing shunt and drain components when these are removed [5]. A series of culture results are summarized in Table 3. Infections caused by *C auris* are a growing concern in healthcare due to their high resistance rates, risk for environmental contamination and nosocomial transmission, and potential for severe outcomes. In the United States, approximately 90% of *C auris* isolates have demonstrated resistance to fluconazole, about 30% to amphotericin B, and less than 5% to echinocandins. Consequently, echinocandins are generally considered the first-line therapy for most *C auris* infections. However, echinocandins exhibit poor penetration into the CNS. Based on CDC recommendations and in vitro studies demonstrating the successful eradication of *C auris* with a combination of antifungal agents, our team decided to administer the liposomal formulation of amphotericin B along with oral flucytosine.

The CNS is protected by the blood-brain barrier, which limits the penetration of many antifungal agents [6]. The selection of the most appropriate antifungal therapy must consider the pharmacological characteristics and pharmacokinetic/pharmacodynamic parameters that impact CNS penetration, such as lipophilicity, protein binding, molecular size, volume of distribution, and efflux pump affinity. The liposomal formulation of amphotericin B has been the cornerstone in treating most fungal CNS infections and is often combined with flucytosine due to its excellent CNS penetration. Flucytosine is typically administered enterally, which is preferred when the patient has enteral access. This approach eliminates the challenges posed by a regimen employing 2 intravenous agents, 1 of which requires infusion over 1–2 hours. However, if the patient lacks enteral access, this route becomes challenging, although several studies suggest that a 50 mg/mL or 10 mg/mL oral suspension of flucytosine can be compounded by opening the capsules and mixing the contents with either OraPlus and OraSweet or distilled water [7, 8]. Despite this, many inpatient hospitals may face limitations in compounding due to sterility concerns and may need to rely on outsourcing to a compounding facility.

A case report by Singhal et al details the successful management of *C auris* CNS shunt infection using intraventricular caspofungin [9]. The patient initially received treatment with liposomal amphotericin B and flucytosine; however, following the identification of *C auris* from a shunt tap within 48 hours, the treatment regimen was adjusted to include intravenous caspofungin in combination with flucytosine. Due to persistently positive extraventricular drain fluid cultures, the therapeutic approach was further modified 6 days later, with the introduction of intraventricular caspofungin at a dose of 10 mg daily, after obtaining informed consent. The regimen also continued to include systemic caspofungin and flucytosine, with the addition of oral voriconazole. The IDSA practice guidelines recommend considering intraventricular antimicrobial therapy for patients with healthcare-associated ventriculitis and meningitis that respond poorly to systemic antimicrobial therapy alone [5]. While intraventricular administration of antifungal agents may be necessary in specific cases, this approach carries risks, including intraparenchymal hemorrhage with intraventricular extension [10]. Additionally, intraventricular administration has not been associated with a statistically significant increase in the rate of clinical cure, warranting careful evaluation of its use [11].

In 2016, hospitals and healthcare facilities in New York reported numerous clinical and surveillance cases of *C auris* [12]. Investigations into pan-resistant *C auris* were conducted to assess its susceptibility to combinations of currently available antifungal drugs. *Candida auris* surveillance and clinical samples from blood or urine were tested using selective culture media, and fungal isolates were identified by matrix-assisted laser desorption/ionization–time of flight mass spectrometry and Sanger sequencing of ribosomal ITS2 genes. Conventional antifungal susceptibility testing with single drugs was done according to the Clinical and Laboratory Standards Institute M-60A method, and susceptibility breakpoints were interpreted per CDC recommendations [13, 14]. Fixed concentration of 2-drug combinations was tested in custom-made plates from Trek Diagnostics System. They found that these pan-resistant *C auris* strains were 100% inhibited in vitro by combinations of 2 antifungal drugs using fixed concentrations achievable in vivo and that flucytosine combinations with either amphotericin B, azoles, or echinocandins were the most effective.

This case report contributes to the understanding of effective treatment strategies for *C auris* ventriculitis, highlighting the role of liposomal amphotericin B and flucytosine in achieving favorable outcomes without resorting to intraventricular administration of antifungal agents. Prompt identification, appropriate antifungal therapy, and stringent infection control measures as recommended by the CDC are crucial in managing *C auris* infections [15, 16]. At our institution, screening for *C auris* colonization status using PCR is routine for patients transferred from an SNF or LTACF, which aids us in timely determination of isolation needs. In addition, the PCR test result was instrumental in suggesting appropriate empiric treatment of ventriculitis infection in this case.

## CONCLUSIONS

*Candida auris* poses a significant threat in healthcare settings due to its multidrug resistance. This case underscores the importance of tailored treatment approaches in managing *C auris* ventriculitis. Continued vigilance and infection control measures are essential in mitigating the spread of *C auris* and improving patient outcomes. Treatment guidelines on effective treatment in various types of cultures such as blood and CSF as well as aggressive and consistently practiced infection control measures in healthcare settings are imperative to prevent transmission of *C auris*.

## Notes

***Acknowledgments.*** All authors assisted with drafting and editing of the manuscript, have reviewed the final version to be published, and agree to be accountable for all aspects of the work.

***Patient consent.*** The patient's written consent was obtained, and the design of the work has been approved by local research committees.

***Potential conflicts of interest***. All authors: No reported conflicts.
